# Hyperoxidized PRDX3 as a specific ferroptosis marker

**DOI:** 10.1093/lifemeta/load042

**Published:** 2023-11-18

**Authors:** Yuelong Yan, Boyi Gan

**Affiliations:** 1 Department of Experimental Radiation Oncology, The University of Texas MD Anderson Cancer Center, Houston, TX 77030, USA; 2 The University of Texas MD Anderson UTHealth Graduate School of Biomedical Sciences, Houston, TX77030, USA

**Keywords:** Ferroptosis, biomarker, PRDX3, hyperoxidation, lipid peroxidation

## Abstract

The lack of a reliable and specific marker for ferroptosis has hindered the advancement of treatments related to this cell death mechanism toward clinical application. A recent study published in *Molecular Cell* has identified hyperoxidized peroxiredoxin 3 (PRDX3) as a promising marker for ferroptosis, opening up new avenues for monitoring and targeting ferroptosis in disease treatment.

Ferroptosis represents a form of regulated cell death driven by disturbed iron metabolism and the excessive accumulation of detrimental lipid peroxides on cellular membranes [1]. It is distinctive from other forms of regulated cell death, such as apoptosis, necroptosis, and pyroptosis, through its unique morphological, biochemical, and genetic characteristics [2]. To safeguard against the adverse effects of lipid peroxides, mammalian cells have evolved robust antioxidant systems. The principal defense mechanism against ferroptosis is the solute carrier family 7 member 11 (SLC7A11)-cyst(e)ine-glutathione (GSH)-glutathione peroxidase 4 (GPX4) axis (Fig. 1) [3]. Within this signaling axis, SLC7A11 plays a pivotal role in importing extracellular cystine, which is subsequently reduced to cysteine to synthesize intracellular GSH [4]. GPX4, acting as a phospholipid hydroperoxidase, utilizes GSH as a cofactor to catalyze the conversion of lipid peroxides into non-toxic lipid alcohols, effectively suppressing ferroptosis (Fig. 1) [3]. A variety of ferroptosis inducers (FINS) have been identified, capable of triggering potent ferroptosis in cancer cells by inhibiting either GPX4 (as exemplified by RAS-selective lethal 3 (RSL3)) or the SLC7A11-mediated cystine uptake (such as with erastin). These FINS hold significant promise in the treatment of specific types of cancer. Conversely, ferroptosis inhibitors have shown potential in treating diseases associated with excessive ferroptosis, such as ischemic organ injuries, and various degenerative diseases [2, 3].

To effectively translate ferroptosis-related treatments into clinical application, it is imperative to assess the occurrence of ferroptosis under specific pathological and physiological contexts; consequently, the discovery of markers capable of precisely identifying cells undergoing ferroptosis holds significant value. However, the current lack of established biomarkers, equivalent to cleaved caspase-3 for apoptosis detection, presents a significant hurdle in the precise identification of ferroptosis, especially in tissues. For example, malondialdehyde (MDA) and 4-hydroxynonenal (4-HNE), byproducts of lipid peroxidation, are frequently utilized as ferroptosis markers [2, 3]. Nevertheless, these secondary products of lipid peroxidation can also arise from other oxidative stress conditions unrelated to ferroptosis. Hence, the quest for a specific ferroptosis biomarker remains a challenging endeavor within the field of ferroptosis research. A recent study by Cui *et al*. may hold the key to finally identifying the long-sought ferroptosis marker, enabling a more precise examination of this unique form of cell death [5].

Peroxiredoxins (PRDXs) constitute a family of cysteine-dependent peroxidase enzymes known for their important role in cellular redox regulation. These enzymes can undergo a specific posttranslational modification referred to as hyperoxidation, utilizing peroxides as substrates [6]. Given that ferroptosis is primarily instigated by the excessive accumulation of lipid peroxides, the authors embarked on a study to investigate the oxidation status of PRDXs during ferroptosis. Their findings were particularly noteworthy, as they revealed that PRDX3 undergoes hyperoxidation in response to various FIN treatments. Remarkably, this hyperoxidation did not occur when other stimuli, triggering other types of cell death, were applied. These intriguing observations, derived from experiments conducted across multiple cancer cell lines, pointed to hyperoxidized PRDX3 as a potential specific marker for ferroptosis [5].

The research further delved into the potential role of PRDX3, which is primarily localized in the mitochondria, in regulating ferroptosis. It was observed that, upon hyperoxidation induced by FIN treatment, hyperoxidized PRDX3 translocates from the mitochondria to the plasma membrane, indicating a role of lipid peroxidation within the mitochondria in regulating PRDX3 hyperoxidation during the ferroptotic process. The authors also showed that the deletion of PRDX3 confers heightened resistance to ferroptosis in cancer cells triggered by erastin, a well-established FIN known for its mode of action in blocking cystine uptake. The study also provided important mechanistic insights, revealing that hyperoxidized PRDX3, situated at the plasma membrane, promotes ferroptosis likely by inhibiting cystine uptake (Fig. 1) [5].

To extend the applicability of their research findings into *in vivo* contexts, the authors conducted further investigations involving animal models of alcoholic liver disease (ALD) and nonalcoholic fatty liver disease (NAFLD). These diseases have both been linked to excessive lipid peroxidation [7], although it remained uncertain whether ferroptosis was a contributing factor. In their study, the researchers employed western blotting analysis with an antibody specifically targeting hyperoxidized PRDX3. Their analyses revealed that hyperoxidized PRDX3 was observed in liver lysates from animals suffering from ALD or NAFLD, but not from control animals. Notably, the biomarkers of apoptosis (cleaved caspase-3) and necroptosis (phosphorylated mixed lineage kinase-domain-like protein (p-MLKL)) showed no discernible differences between the control group and the disease models. These findings strongly imply the occurrence of ferroptosis in liver damage associated with ALD and NAFLD, thereby suggesting the potential utility of ferroptosis inhibitors as a viable treatment approach for these diseases [5].

In summary, this research provides valuable insights into the distinctive role of hyperoxidized PRDX3 as a specific marker for ferroptosis and highlights the intricate interplay between mitochondrial lipid peroxidation and PRDX3 hyperoxidation in regulating this form of cell death (Fig. 1). It also underscores the potential therapeutic implications of targeting PRDX3 in the context of ferroptosis-related diseases. Moreover, this study offers additional insights into the search for other ferroptosis-specific markers. Oxidation of cysteine thiols to sulfenic acid, sulfinic acid, and sulfonic acid can occur under ferroptosis conditions, and quantitative reactive cysteinome profiling may be a promising approach to identify oxidized proteins induced by ferroptosis [8]. These oxidized proteins have the potential to serve as markers for ferroptotic cells.

This intriguing study also prompts several important questions for future investigation. Mechanistic inquiries, including the translocation of PRDX3 from mitochondria to the plasma membrane during ferroptosis, the specific subcellular location of PRDX3 hyperoxidation (whether it predominantly occurs in mitochondria or on the plasma membrane), and the precise mechanism by which hyperoxidized PRDX3 inhibits cystine uptake, remain unclarified and present captivating avenues for subsequent studies. In addition, while the authors demonstrate that other cell death modes, such as apoptosis, do not induce hyperoxidation of PRDX3, it is critical to investigate the impact of additional stimuli, such as oxidative stresses that do not induce ferroptosis, on PRDX3 hyperoxidation. These analyses are pivotal in assessing whether this modification genuinely serves as a distinctive marker for ferroptosis. Furthermore, evaluating the utility of this marker in other pathological or therapeutic contexts associated with ferroptosis, such as tumors treated with diverse ferroptosis-inducing therapies like immunotherapy, radiotherapy, and chemotherapy [9], will be of great significance. This research sets the stage for an exciting exploration of ferroptosis and its potential applications in various fields of medicine and biology.

## Figures and Tables

**Figure 1:**
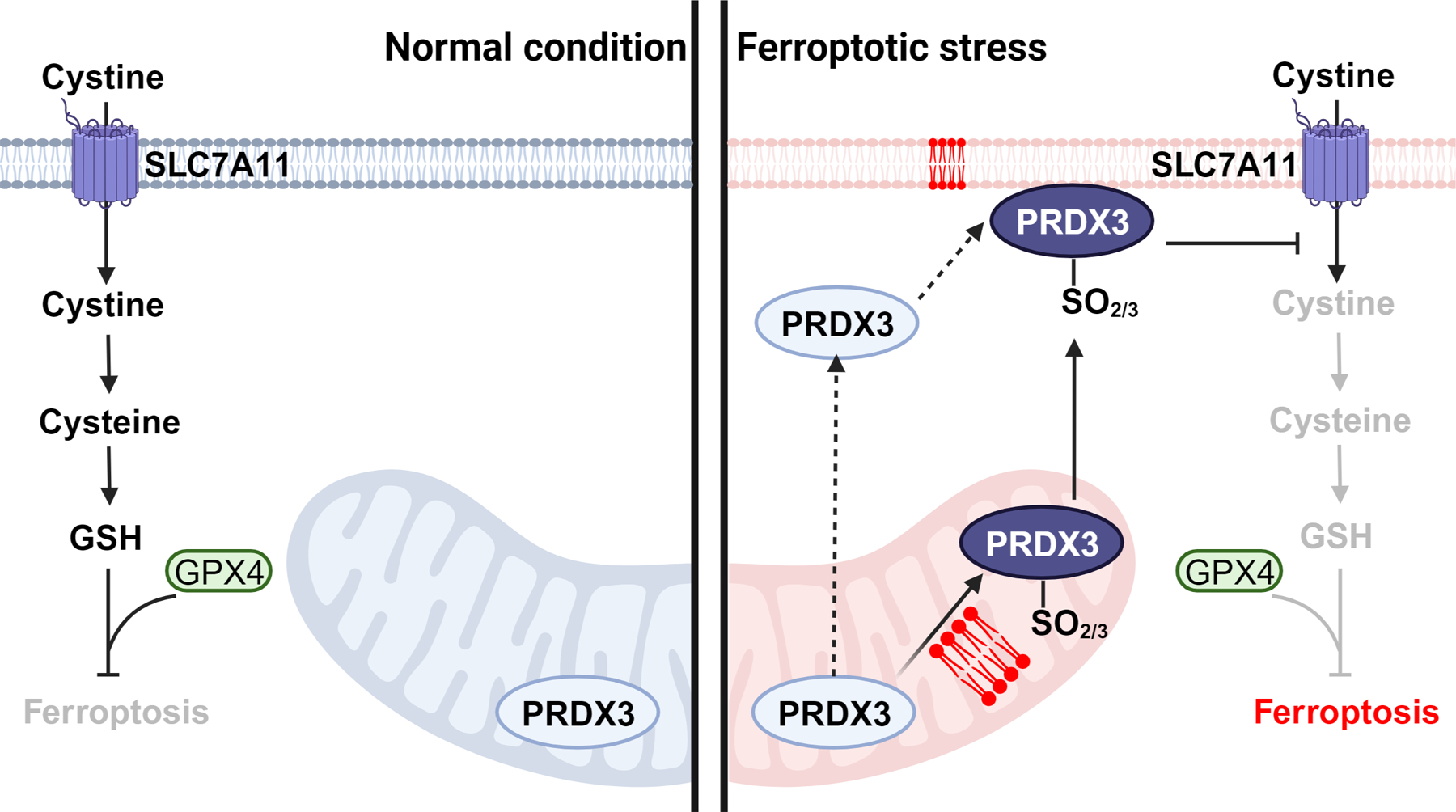
Model depicting the hyperoxidation of PRDX3 during ferroptosis. Left panel: under normal conditions, SLC7A11-mediated cystine uptake promotes GSH biosynthesis. GSH, in turn, serves as a co-factor for GPX4 to effectively detoxify lipid peroxidation and suppress the occurrence of ferroptosis. Right panel: during ferroptotic stress, PRDX3 undergoes hyperoxidation as a result of exposure to mitochondrial lipid peroxides. Subsequently, hyperoxidized PRDX3 is translocated from the mitochondria to the plasma membrane. (It should be noted that an alternative sequence may also occur, where mitochondrial PRDX3 first relocates to the plasma membrane and then undergoes hyperoxidation there. The current study does not provide definitive evidence to distinguish between these two models.) Regardless of the sequence, the presence of hyperoxidized PRDX3 at the plasma membrane inhibits cystine uptake, thereby promoting ferroptosis. Phospholipids in red refer to lipid peroxides on cellular membranes. Abbreviations: PRDX3, peroxiredoxin 3; GPX4: glutathione peroxidase 4; GSH, glutathione; SLC7A11: solute carrier family 7 member 11.
